# Efficacy and tolerability of zuranolone in patients with depression: a meta-analysis of randomized controlled trials

**DOI:** 10.3389/fphar.2023.1334694

**Published:** 2024-01-15

**Authors:** Youjia Qiu, Yuchen Tao, Aojie Duan, Xingzhou Wei, Menghan Wang, Minjia Xie, Zhouqing Chen, Jing Shang, Zhong Wang

**Affiliations:** ^1^ Department of Neurosurgery & Brain and Nerve Research Laboratory, The First Affiliated Hospital of Soochow University, Suzhou, Jiangsu, China; ^2^ Suzhou Medical College of Soochow University, Suzhou, Jiangsu, China; ^3^ Department of Psychiatry, The First Affiliated Hospital of Soochow University, Suzhou, Jiangsu, China

**Keywords:** depression, zuranolone, major depressive disorder, postpartum depression, metaanalysis

## Abstract

**Background:** As a novel antidepressant drug, zuranolone has been initially applied in treating depression. This study investigated the efficacy and safety of its administration in patients with depression.

**Methods:** The Embase, PubMed, and Cochrane library databases were searched for available studies up to 1 Nov 2023. The primary outcome was the change on day 15 depression severity scores compared to baseline. Secondary outcomes included remission and response rates on day 15. Safety outcomes included incidence of treatment-emergent adverse events (TEAEs) and individual AEs. Trial sequential analysis (TSA) was used to evaluate the ideal samplesize.

**Results:** Six studies with 1884 patients were included. Zuranolone offered significantly greater changes in day 15 depression severity scores (mean difference = 2.43, 95% confidence interval [CI]: 1.36 to 3.49, *p* < 0.00001) compared to placebo; this was also observed at other time points. Differences in response (relative risk [RR] = 1.33, 95% CI: 1.15 to 1.54, *p* < 0.0001) and remission (RR = 1.46, 95% CI: 1.15 to 1.85, *p* = 0.002) rates were also statistically significant. For safety outcomes, zuranolone group showed more incidence of TEAE than the placebo group (RR: 1.15, 95% CI: 1.06 to 1.25, *p* = 0.0005, *I* ^*2*^ = 0%). As for individual AEs, significant differences were observed in dizziness (RR = 2.17, 95% CI: 1.22 to 3.86, *p* = 0.008) and somnolence (RR = 2.43, 95% CI: 1.35 to 4.37, *p* = 0.003. No significant difference was observed in other AEs. The result of TSA indicated that the cumulative curve crossed the conventional (Z = 1.96) boundary but not reach TSA boundary (RIS = 1910).

**Conclusion:** Our findings suggest that zuranolone has a rapid short-term antidepressant effect during administration. Although more TEAEs were observed in zuranolone, most of them were slight and temporary. However, studies with larger sample sizes and longer follow-up are needed.

**Systematic Review Registration:** https://inplasy.com/inplasy-2023-5-0104/, identifier INPLASY202350104.

## 1 Introduction

Depression is a highly prevalent mental disorder that causes considerable disability (Rotenstein et al., 2016), and is associated with an increase in suicide rates (Rihmer and Gonda, 2012), a decrease in quality of life (Han et al., 2020), and adverse psychological prognosis (Moussavi et al., 2007) if not treated probably. Major depressive disorder (MDD) is the most common type of depression, it has demonstrated increasing prevalence (Otte et al., 2016), and affects more than 264 million individuals globally (Zhou et al., 2019). In this context, several groups including pregnant women and the elderly are more prone to depression (Lu et al., 2021), women may suffer from postpartum depression (PPD) during the period of pregnancy or childbirth (Gunduz-Bruce et al., 2022). Studies have reported that over 50% of women may suffer from PPD and, the majority do not receive an appropriate diagnosis and adequate treatment, and the condition may persist for years (Vliegen et al., 2014; Netsi et al., 2018), thereby increasing the disease burden and adversely affecting the entire family (Valla et al., 2016). Although antidepressant drugs may be effective in some patients, satisfactory symptom relief is not obtained in most case due to insufficient response, persistent residual symptoms, frequent relapse, or recurrence of symptoms (López-Torres Hidalgo and DEP-EXERCISE Group, 2019). Additionally, traditional antidepressants have certain limitations including unstable efficacy and delayed onset of clinical response (Tartt et al., 2022). It is therefore essential to develop innovative, effective, and safe antidepressant treatments that are capable of offering rapid symptom relief.

Over the past two decades, there have been considerable advances in the understanding on depression. The notion that depression is caused by abnormalities in the amino acid neurotransmitter system, particularly gamma-aminobutyric acid (GABA), has undergone significant changes, notably, the dysregulation of GABAergic signaling has been found to be associated with the onset of MDD (Ali et al., 2023). GABA, the principal inhibitory neurotransmitter in the brain, can affect a variety of brain circuits that are crucial to a range of behavioral states (vigilance, anxiety, sleep, seizures, and memory, among others); its effects are mediated through the GABA_A_ receptor (Health, 2021). Given its crucial role in the function of neural circuits, a variety of therapeutically significant medications including benzodiazepines, barbiturates, and anesthetics, target GABA_A_ receptors. In this context, Hecking et al. found the plasma and cerebrospinal fluid levels of GABA to be lowered in patients with depression; this indicated the potential role of GABA in the pathogenesis of depression (Hecking et al., 2021). Notably, it is currently believed that PPD may be triggered by a rapid decline in neurosteroid levels during childbirth. Allopregnanolone, the neuroactive metabolite of progesterone, is a powerful modulator of GABA_A_ receptors, the primary inhibitory receptor in brain (Patterson et al., 2024). This suggests that MDD and PPD may have shared pathophysiological elements (Gunduz-Bruce et al., 2019); newly developed antidepressants targeting the GABAergic system have therefore been used in clinical practice. For instance, brexanolone, a positive allosteric modulator of the GABA_A_ receptors, has been demonstrated to have rapid antidepressant effect in PPD and MDD (Uzunova et al., 1998; Zheng et al., 2019). However, the insignificant long-term antidepressant efficacy, impatient hospitalization, frequent long-time injections, requirements of monitoring sedation and unconsciousness may restrict the clinical application (Zheng et al., 2019). Similar to brexanolone, zuranolone (previously known as SAGE-217) is an allosteric modulator of the GABA receptor that acts by enhancing receptor activity; this could help regulate anxiety, mood, and other neurological functions.

Several reviews have evaluated ongoing and completed studies in zuranolone (Walkery et al., 2021). However, meta-analyses that comprehensively analyzed the antidepressant effect of zuranolone are lacking. This meta-analysis was therefore performed to evaluate the outcomes of oral zuranolone administration in patients with depression.

## 2 Materials and methods

This meta-analysis was performed according to the criteria of the Preferred Reporting Items for Systematic Reviews and Meta-Analysis (PRISMA) statement (Liberati et al., 2009). A formal protocol was developed and registered on an international platform for registration of systematic review and meta-analysis protocols (INPLASY number: 202350104).

### 2.1 Search strategy

To find eligible studies, a comprehensive search of literature was performed across the PubMed, Cochrane, and Embase databases up to 1 Nov 2023. The following keywords were included for the search: zuranolone, SAGE-217 (another name of zuranolone used in earlier clinical trial), depression, MDD, major depressive disorder, PPD, and postpartum depression. The search strategy included both MeSH and free terms; the description of search results are shown in Supplementary Table S1. To ensure comprehensiveness of the search, reviews and meta-analyses were also read and correlated during data extraction.

### 2.2 Eligibility

The following inclusion criteria were based on the PRISMA statement: a) population: patients aged over 18 years with a diagnosis of MDD or PPD according to the Diagnostic and Statistical Manual of Mental Disorders third, fourth, or fifth edition, or International Classification of Diseases 10th edition; b) intervention: patients who received different dosages (e.g., 20 mg/d, 30 mg/d, and 50 mg/d) of oral zuranolone without another antidepressant; c) comparison: patients who received placebo treatment; d) outcome: primary outcome was change in depression severity scores from baseline at day 15. Long-time observations on day 42 and day 45 were considered the same time observations. Secondary efficacy outcomes included response (defined as 50% reduction of 17-item Hamilton rating scale for depression [HAMD-17] score from baseline) and remission (defined as HAMD-17 scores ≤ 7) (Hamilton, 1960); safety outcomes were total adverse events (AEs), the individual AEs, and withdraw rates; e) study design: only randomized controlled trials (RCTs) were enrolled for further analysis.

Studies fulfilling any one of the following criteria were excluded: a) study type: case report or series, editorial, cohort studies, review, letter, or comment; b) language: articles not written in English; c) other treatments used for combined intervention; and d) non-availability of depression evaluation results.

### 2.3 Study selection and data collection

Titles and abstracts for each study were screened and selected by two independent reviewers (YJQ and YCT). Discrepancies were resolved by an independent reviewer (AJD) who did not participate in the procedure. The obtained data included the study design, authors names, year, essential characteristics of the included patients, exclusion and inclusion criteria, diagnostic criteria for MDD, study duration, outcome indicators, and sample size.

### 2.4 Risk of bias and quality assessment

Risk of bias was assessed using the Cochrane Collaboration tool by two independent reviewers (MHW and XZW), which evaluated six domains (Higgins et al., 2011). R software (version 4.3.0) was used to assess whether each index had low, unclear, or high risk of bias. The certainty of evidence of enrolled studies was evaluated based on the Grading of Recommendations Assessment, Development, and Evaluation scale (Guyatt et al., 2023). Discrepancies were resolved through discussion.

### 2.5 Statistical analysis

Estimates of differences for dichotomous and continuous variables have been presented as the relative risk (RR) and mean differences (MD) respectively, 95% confidence intervals (CI) were calculated using random-effect model of the Mantel Haenszel method (Mantel and Haenszel, 1959). In case where the extracted data described continuous variables as medians, interquartile ranges, or ranges instead of the mean and standard deviation, these data were transformed by the method used by Hozo et al. (2005). The Cochran Q and I^2^ tests were used to assess the heterogeneity of the included studies (www.training.cochrane.org/handbook.). *I* ^*2*^ values of > and < 50% were considered to indicate high and low heterogeneity, respectively (Borenstein et al., 2017). Sensitivity analysis was performed by removing one study in turn to explore its influence on heterogeneity. Meta-regression and subgroup analysis were performed for different types of depression and oral dosages to detect the source of heterogeneity. Two-tailed tests were performed in all cases, and *p* < 0.05 was considered statistically significant; all analyses were performed using RevMan 5.3.4 Software.

Trial sequential analysis (TSA) was used to evaluate type I and II errors induced by the limited sample size. The results were represented by a Cartesian graph with the sample size on the *x*-axis and the cumulative Z-score on the *y*-axis. Two lines parallel to the *x*-axis represented the conventional lines of statistical significance. The required information size (RIS) was auto-estimated, this represented the appropriate sample size. The risks for type I and type II errors were set at 5% and 20%, respectively. In case where the cumulative Z curve crossed the conventional and TSA boundaries, the results were considered to be robust (eliminating the need for any further studies).

## 3 Results

### 3.1 Search results

**FIGURE 1:**
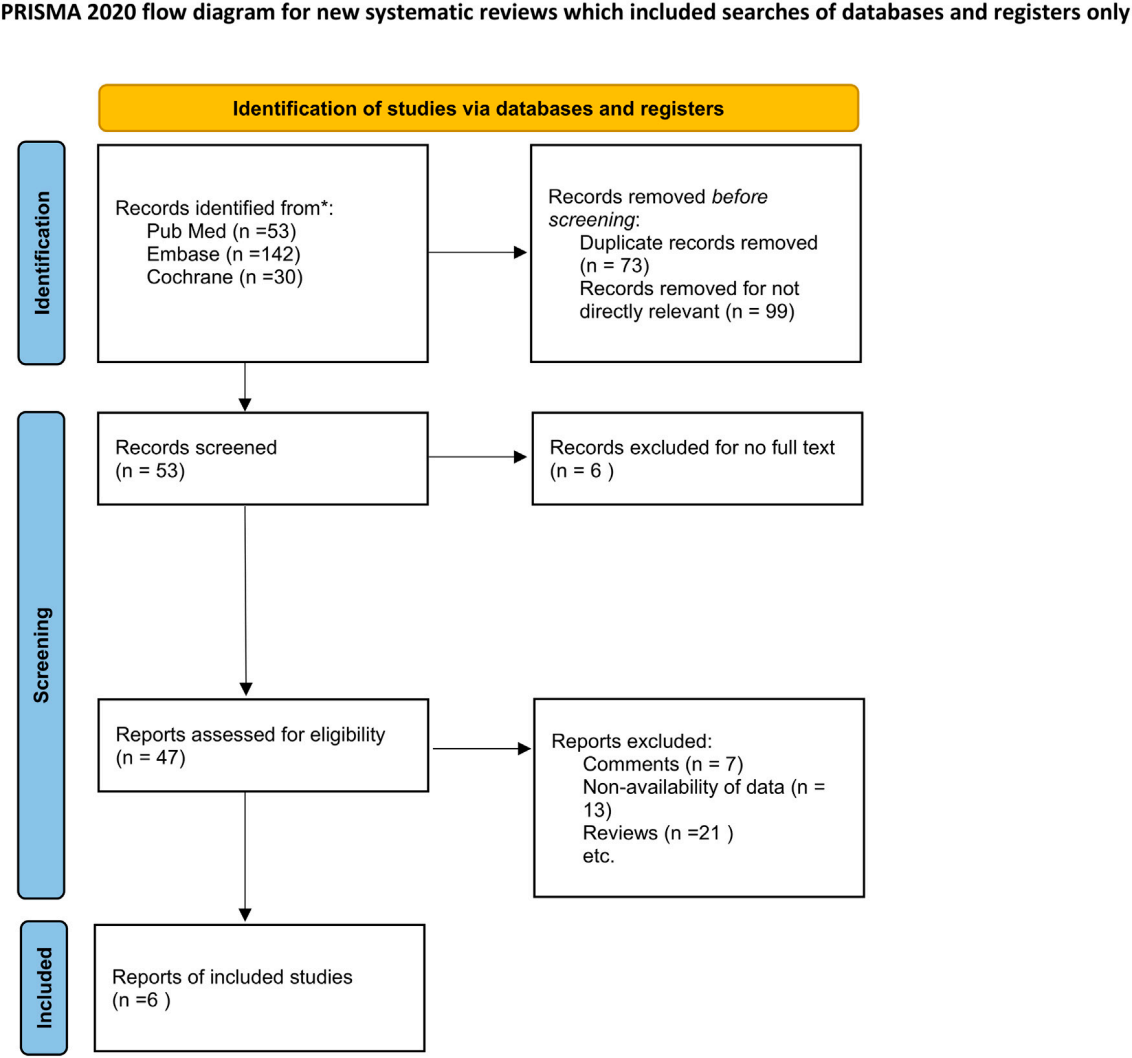
PRISMA flowchart of study identification and selection process.

Overall, 225 titles and abstracts were searched for eligibility from PubMed, Embase and the Cochrane library. A total of 53 studies were excluded due to duplicate publication and 172 records were removed as they were not directly relevant to the topic of our study. The full text of 47 studies were then read for eligibility; 13 of these were excluded owing to non-availability of data. In addition, 7 comments and 21 reviews were excluded as they were not RCTs. Six RCTs (Gunduz-Bruce et al., 2019; Deligiannidis et al., 2021; Clayton et al., 2023a; Clayton et al., 2023b; Deligiannidis et al., 2023; Kato et al., 2023)were therefore finally included in the meta-analysis. The details pertaining to the screening results are shown in Figure 1.

### 3.2 Baseline characteristics

A total of 1884 patients with MDD or PDD were included from four RCTs. Basic information pertaining to each enrolled study is shown in Table 1; 1,341 (71.2%) patients were female and the mean age of the participants was 37.85 ± 12.18 years. The duration of oral medication ranged from 1 to 14 days, and that of follow-up extended to 45 days. The dosage of oral zuranolone was 20 mg, 30 mg or 50 mg once a day. In addition to differences in drug dosage, there were also variations in the types of depression; Three studies reported outcomes in MDD, while two studies reported those in PDD. No statistical difference was observed between the zuranolone and placebo groups in terms of baseline HAMD scores (MD: −0.02, 95% CI: −0.11 to 0.07, *p* = 0.62) (Supplementary Figure S1).

**TABLE 1:** Characteristics and outcome measures of the included trials.

	Number of patients	Dose(mg)	Administration	Age, mean (sd)		Female sex—no. (%)		Baseline HMAD, mean (sd)		Outcome measures	Depression type
				Zuranolone	Placebo	Zuranolone	Placebo	Zuranolone	Placebo		
Clayton (2023b)	581	20	Oral, once-daily, 14-day	41.9 (12.2)	41.4 (12.2)	112 (70.4)	106 (67.5)	25.9 (2.9)	25.8 (3.1)	Score changes in HDRS-17, CFI-S; Response (>50% reduction in HDRS-17); Remission (HDRS-17 < 7)	MDD
		30	Oral, once-daily, 14-day	42.3 (11.8)	41.4 (12.2)	121 (72.9)	106 (67.5)	25.9 (2.9)	25.8 (3.1)		
Deligiannidis (2021)	150	30	Oral, once-daily, 14-day	29.3 (5.4)	27.4 (5.3)	76 (100)	74 (100)	28.4 (2)	28.8 (2)	Score changes in HAMD-17, MADRS, HAM-A, CGI-I; Response (>50% reduction in HAMD-17); Remission (HAM-D ≤ 7)	PPD
Gunduz-Bruce (2019)	89	30	Oral, once-daily, 14-day	49.1 (13.6)	38.3 (12.2)	25 (56)	30 (68)	25.2 (2.6)	25.7 (2.4)	Score changes in HAMD-17, MADRS and CGI; Response (>50% reduction in HAMD-17); Remission (HAM-D ≤ 7)	MDD
Kato (2023)	249	20	Oral, once-daily, 14-day	39.3 (12.6)	40.8 (1 0.6)	49 (57.6)	47 (57. 3)	24.8 (2.4)	24.5 (2.1)	Score changes in HAMD-17, MADRS and CGI; Response (>50% reduction in HAMD-17); Remission (HAM-D ≤ 7)	MDD
		30	Oral, once-daily, 14-day	38.8 (1 2.0)	40.8 (1 0.6)	47 (57.3)	47 (57. 3)	24.6 (2.2)	24.5 (2.1)		
Deligiannidis (2023)	196	50	Oral, once-daily, 14-day	30.0 (5.9)	31.0 (6.0)	98 (100)	98 (100)	28.6 (2.5)	28.8 (2.3)	Score changes in HAMD-15, CGI MADRS and HAM-A; Response (>50% reduction in HAMD-15); Remission (HAM-D ≤ 7)	PPD
Clayton (2023a)	537	50	Oral, once-daily, 14-day	38.4 (12.3)	40.1 (12.6)	186 (69.4)	166 (61.7)	26.8 (2.6)	26.9 (2.7)	Score changes in HAMD-15, CGI, MADRS and HAM-A; Response (>50% reduction in HAMD-15); Remission (HAM-D ≤ 7)	MDD

### 3.3 Effective outcomes

**FIGURE 2:**
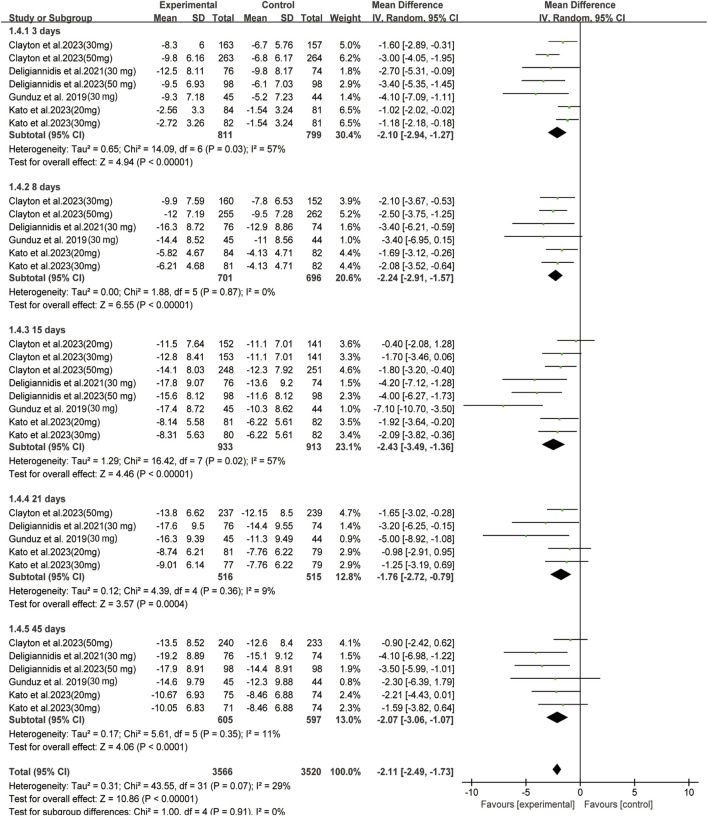
Forest plot for changes in HAMD-17 score in depressive patients after received oral zuranolone or placebo treatment at different time points.

**FIGURE 3:**
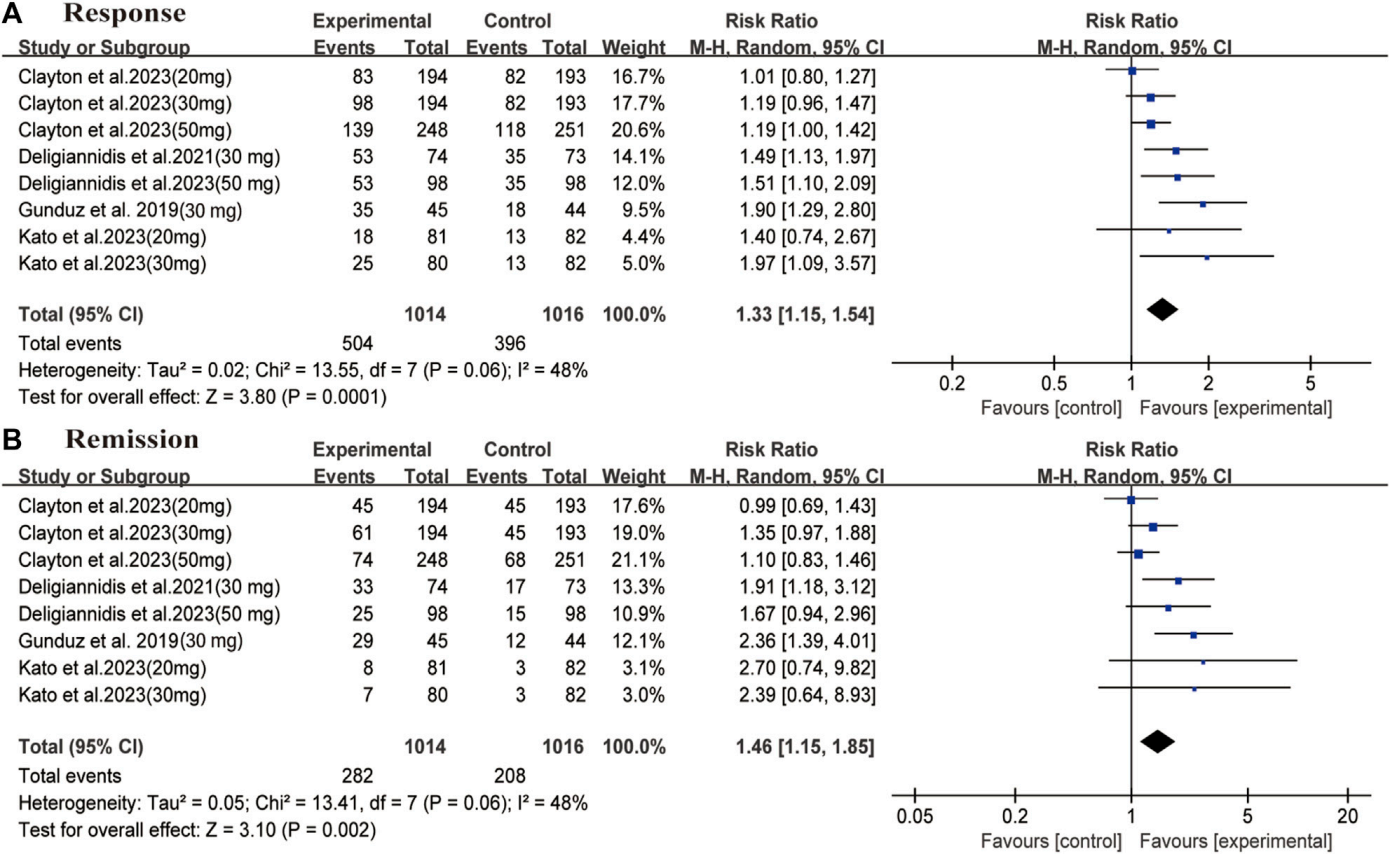
Forest plot for differences of the zuranolone group and the placebo group in **(A)** response and **(B)** remission.

In all enrolled studies, HAMD-17 score served as the primary indicator in evaluating improvement of depressive symptoms. A statistically significant difference was observed between the two groups in terms of changes in day 15 depression severity scores (MD: −2.43, 95% CI: −3.49 to −1.36, *p* < 0.00001, *I* ^*2*^ = 57%). The changes between the two groups were also compared at different time intervals; significant differences were observed on day 3 (MD: −2.10, 95% CI: −2.94 to −1.27, *p* < 0.00001, *I* ^*2*^ = 57%), 8 (MD: −2.24, 95% CI: −2.91 to −1.57, *p* < 0.00001, *I* ^*2*^ = 0%), 21(MD: −1.76, 95% CI: −2.72 to −0.79, *p* = 0.0004, *I* ^*2*^ = 9%), and 45 (MD: −2.07, 95% CI: −3.06 to −1.07, *p* < 0.0001, *I* ^*2*^ = 11%) (Figure 2). Regarding secondary outcomes, the zuranolone group also demonstrated significant improvements in terms of the response (RR = 1.33, 95% CI: 1.15 to 1.54, *p* = 0.0001) and remission (RR = 1.46, 95% CI: 1.15to 1.85, *p* = 0.002) rates (Figure 3).

### 3.4 Safety outcomes

**FIGURE 4:**
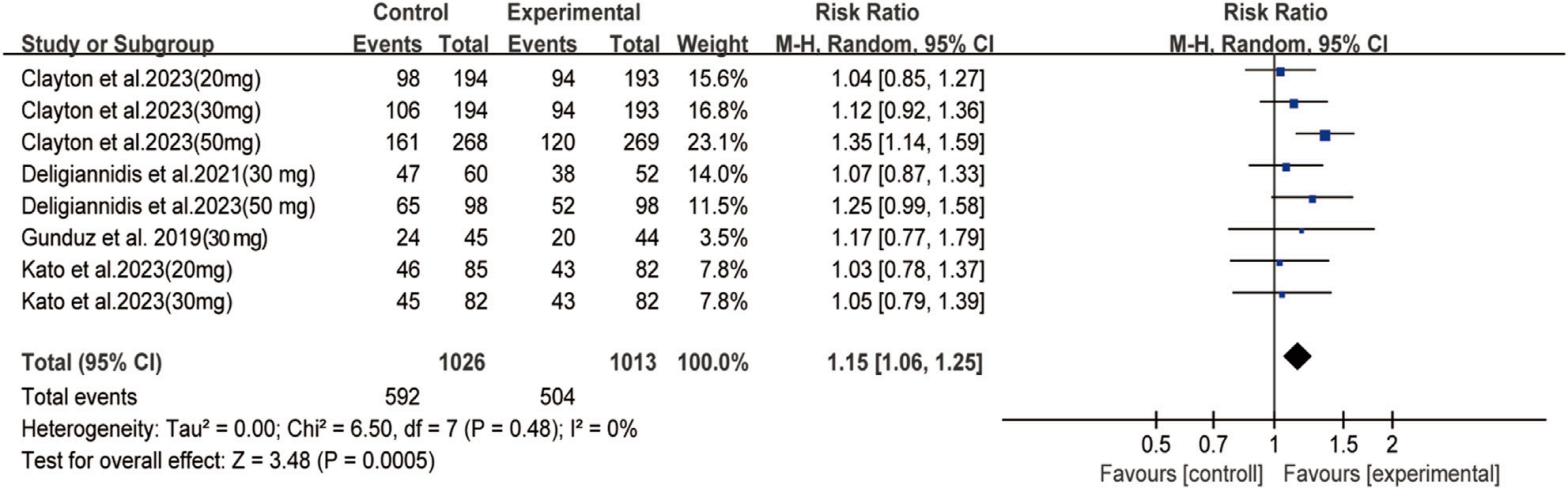
Forest plot for differences of the zuranolone group and the placebo group in TEAE.

Among depressive patients who received oral zuranolone, no deaths were observed during the treatment period. Significant differences were observed between the zuranolone and placebo groups in terms of TEAE (RR: 1.15, 95% CI: 1.06 to 1.25, *p* = 0.0005, *I* ^*2*^ = 0%) (Figure 4), the most common AEs were headache, dizziness, and nausea. No significant difference was observed in terms of common AEs, except for dizziness (RR = 2.17, 95% CI: 1.22 to 3.86, *p* = 0.008) and somnolence (RR = 2.43, 95% CI: 1.35 to 4.37, *p* = 0.003) (Supplementary Figures S2, S3). However, there was no difference in withdraw rates between the two groups (Supplementary Figure S4). The detailed information of AEs in each study is shown in Supplementary Figure S4.

### 3.5 Subgroup analysis

Subgroup analysis was performed based on the different dosages of zuranolone on day 15. In the 20 mg group, no statistical difference was observed between the groups in terms of day 15 outcomes (MD: −1.15, 95% CI: −2.64 to 0.34, *p* = 0.13). In both 30 mg and 50 mg group, significant differences were observed between the groups in terms of day 15 outcomes (30 mg: MD: −3.28, 95% CI: −5.25 to −1.32, *p* = 0.001, *I* ^*2*^ = 65%; 50 mg: MD: −2.71, 95% CI: −4.83 to −0.59, *p* = 0.01, *I* ^*2*^ = 62%). A similar trend was observed for response (30 mg: RR: 1.50, 95% CI: 1.18 to 1.90, *p* = 0.0008, *I* ^*2*^ = 51%; 50 mg: MD: 1.29, 95% CI: 1.03 to 1.61, *p* = 0.02, *I* ^*2*^ = 40%) rates in 30 mg and 50 mg zuranolone group, and remission (RR: 1.74, 95% CI: 1.30 to 2.32, *p* = 0.0002) rates in 30 mg zuranolone group (Supplementary Figure S5).

The difference in efficacy was also compared between patients with in MDD and PPD, the major findings are shown in Supplementary Figure S6. In patients with MDD, significant differences were observed between the zuranolone and placebo groups on days 3 (MD: −2.16, 95% CI: −3.28 to −1.04, *p* = 0.0002, *I* ^*2*^ = 64%), 8 (MD: −2.32, 95% CI: −3.11 to −1.53, *p* < 0.00001, *I* ^*2*^ = 0%), 15 (MD: −2.53, 95% CI: −4.08 to −0.99, *p* = 0.001, *I* ^*2*^ = 61%), and 21 (MD: −1.90, 95% CI: −3.34 to −0.47, *p* = 0.009, *I* ^*2*^ = 31%). However, no statistical difference was observed on day 45 (MD: −1.22, 95% CI: −2.43 to −0.02, *p* = 0.05, *I* ^*2*^ = 0%). And for PPD, significant differences were also observed between the zuranolone and placebo groups on days 3 (MD: −3.15, 95% CI: −4.71 to −1.58, *p* < 0.0001, *I* ^*2*^ = 0%), 15 (MD: −4.08, 95% CI: −5.87 to −2.28, *p* < 0.00001, *I* ^*2*^ = 0%). And differ to MDD, statistical difference was observed on day 45 (MD: −3.76, 95% CI: −5.64 to −1.87, *p* < 0.0001, *I* ^*2*^ = 0%). The findings also demonstrated significant reduction in depression severity scores at all time points (Supplementary Figure S6). Regarding secondary outcomes, significant improvement in response was observed in both MDD and PPD cases (MDD: RR: 1.38, 95% CI: 1.10 to 1.72, *p* = 0.005, *I* ^*2*^ = 58%; PPD: RR: 1.50, 95% CI: 1.22 to 1.86, *p* = 0.0002, *I* ^*2*^ = 0%); similar findings were observed for remission at day 15 (MDD: RR: 1.47, 95% CI: 1.04 to 2.09, *p* = 0.07, *I* ^*2*^ = 58%; PPD: RR: 1.81, 95% CI: 1.25 to 2.62, *p* = 0.002, *I* ^*2*^ = 0%) (Supplementary Figure S7).

### 3.6 Heterogeneity and sensitivity analysis

For the primary outcome, high heterogeneity was observed in terms of day 15 outcomes, response, and remission. As shown in Supplementary Figure S8, the results were robust and no heterogeneity was found during sensitivity analysis. Meta-regression was subsequently performed to detect the source of heterogeneity; studies were divided into two groups based on dose and type of depression. However, all statistical parameters demonstrated a *p*-value of > 0.05, indicating no source of heterogeneity in groups receiving different doses and having different types of depression. The detailed results of meta-regression have been presented in Supplementary Table S2.

### 3.7 Risk of bias and quality assessment

**FIGURE 5:**
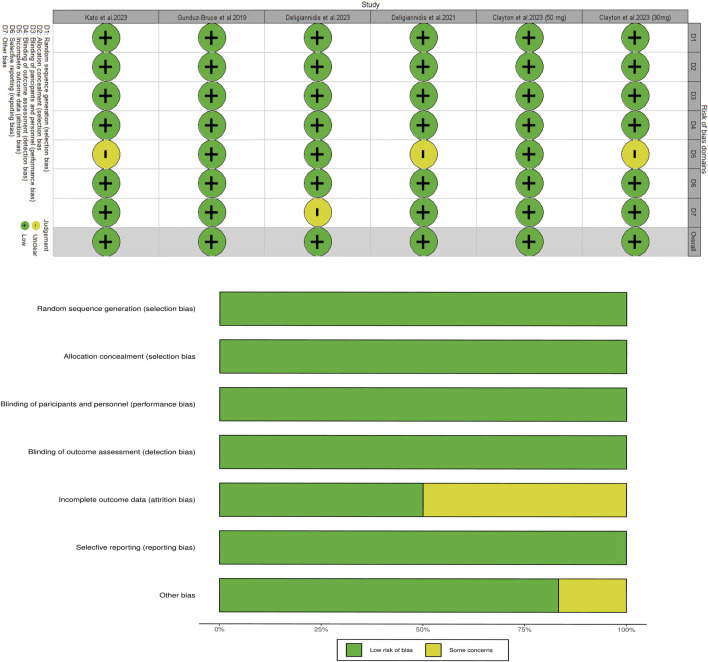
Risk of bias for each study.

The risk of bias across all enrolled RCTs has been shown in Figure 5; the data indicates that all studies were evaluated to be low-risk in most domains. According to the Grading of Recommendations Assessment, Development, and Evaluation scale, the quality of evidence from the RCTs was relatively high (Supplementary Table S3).

### 3.8 TSA

Changes in depression severity scores were used for estimation of sample size. As illustrated in Supplementary Figure S9, the cumulative curve crossed the conventional (Z = 1.96) boundary but not reach TSA boundary (RIS = 1910). In addition, penalized test of TSA also crossed the conventional boundary (Supplementary Figure S10); this indicated that the rapid efficacy of zuranolone has been confirmed, while the sample size from the included RCTs did not reach the ideal value. Further studies with larger sample size are therefore required to validate the reliability of the drug.

## 4 Discussion

To the best of our knowledge, this is the first meta-analysis to evaluate the therapeutic efficacy and tolerability of zuranolone in patients with depression. Based on the analysis of existing clinical trials, zuranolone appears to demonstrate a rapid-onset short-term effect along with a definite sustained effect in depression. Subgroup analysis also confirmed its efficacy in MDD and PPD. On comparison of different dosages, the 30 mg dose showed significantly greater efficacy than the 20 mg dose. The absence of any deaths or significant adverse effects among the 1884 patients from the included studies indicated that zuranolone was well-tolerated during administration.

In our study, significant difference was observed in terms of reduction in day 15 HAMD-17 scores in the zuranolone group; this was indicative of considerable efficacy in rapid control of depressive episodes. Notably, the enrolled trials consistently applied change of HAMD-17 score on day 15 as the primary outcome, which has been recognized as clinically meaningful and substantial improved (Rush et al., 2021). In the study conducted by Clayton et al., the zuranolone group did not achieve this ideal outcome on day 15. However, the *post hoc* analysis showed that approximately 9% patients had no measurable drug concentrations. After excluding these patients, significant difference was observed on day 15 (Clayton et al., 2023a).

In addition, significant differences were observed at other time points. In general, zuranolone exhibited antidepressant effect on day 3 and sustained until day 45. However, the real long-term efficacy of zuranolone requires to be discussed because the results were influenced by the data from a forest plot of two included studies (performed by Deligiannidis et al. on PPD) (Ten Doesschate et al., 2021; Arnaud and Bonthapally, 2022; Ten Doesschate et al., 2022). For MDD, no statistical difference was observed on day 45; two phase III studies (MOUNTAIN and WATERFALL) also showed no statistically significant difference on day 42 (Ten Doesschate et al., 2022). However, Clayton et al. suggested the existence of a robust placebo response as their study involved a large number of visits (Clayton et al., 2023a). This indicates that rapid supervision and assessment may contribute to a placebo response (Khin et al., 2011; Khan et al., 2017). The study also showed a clinically meaningful Cohen d value on *post hoc* analysis, this warranted further exploration of higher dosages, as there was a trend toward improvement (Clayton et al., 2023a). Although the assessment of zuranolone and its practical application in MDD is challenging (Ali et al., 2023), we speculate that its long-term efficacy may be confirmed in future clinical trials, with larger sample sizes, and a relatively higher dosage of administration. In this context, TSA in the present study showed the number of enrolled participants and studies to be inadequate.

Response and remission are the most widely used indicators in studies on depression (Riedel et al., 2010). In another study, the zuranolone group demonstrated greater response and remission on day 15 compared to the placebo group, the findings demonstrated its rapid antidepressant effect (Gunduz-Bruce et al., 2019). Compared to MDD, PPD demonstrated a more positive response to zuranolone at every time point. Notably, a network meta-analysis also demonstrated a high surface under the cumulative ranking curve value for zuranolone (Zhang et al., 2022). This may be attributed to the fact that GABA_A_ receptors cannot adapt to changes during childbirth due to the sharp decline in plasma allopregnanolone concentrations after delivery (Kanes et al., 2017). Zuranolone, as an allopregnanolone antagonist, may restore plasma allopregnanolone concentrations and reset neural networks, thereby improving depressive symptoms in PPD (Kanes et al., 2017). In this context, the response and remission rates with zuranolone exceeded those observed with traditional antidepressants (∼50% and ∼30%, respectively), this indicated the potential advantages (compared to traditional drugs) in terms of achieving rapid antidepressant effect (Deligiannidis et al., 2021; Walkery et al., 2021). Notably, the number of studies evaluating zuranolone in PPD is limited, despite its confirmed efficacy on long and short-term observation (Deligiannidis et al., 2021). In this context, an ongoing double-blinded RCT is evaluating its efficacy in severe PPD (NCT04442503), which may bring novel insights of zuranolone medication in PPD.

Regarding different dosages, 30 mg zuranolone demonstrated statistically significant differences in the reduction of HAMD-17 scores, however, the 20 mg group showed no significant differences. The true efficacy of lower doses of zuranolone in depression warrants be further confirmation, as only two studies has reported the outcomes (Clayton et al., 2023a; Kato et al., 2023). On comparing the data from studies using doses of 20 mg per day and 30 mg per day (which are the only available data), the dose of 30 mg per day appeared to induce a more rapid response and greater reduction in depressive symptoms. Notably, other dosages have been explored in clinical trials. For instance, the WATERFALL was a double blinded RCT which compared zuranolone (50 mg) to placebo in adult participants with MDD. The group receiving zuranolone showed significantly greater improvement on day 15 depressive symptoms compared with that receiving placebo; this confirmed the antidepressant efficacy of a higher dosage of zuranolone. An open-label, 1-year longitudinal study (NCT03864614) is evaluating repeat dosing with zuranolone, and is comparing the efficacy of a dose of 30 mg with that of an increased dose (50 mg) zuranolone in MDD (TherapeuticsSageB, 2020; Walkery et al., 2021). In this context, higher doses of 50 mg have been shown to offer higher response and remission rates compared to a dose of 30 mg (Health, 2021). A study showed the mean reduction in HAMD-17 scores to be 15.9 in the 50 mg group, and 14.9 in the 30 mg group (Health, 2021), indicating that a higher dosage within a safe range may potentially yield greater therapeutic effects.

Regarding safety outcomes, there were no reported deaths among the participants in the included studies. In addition, none of the studies reported increased suicidal ideation after administration (Health, 2021; Clayton et al., 2023a). The most reported AEs were headache, somnolence and dizziness, and there were no statistically significant differences between the two groups in terms of the incidence of most AEs. Although statistically significant differences were observed in terms of dizziness and somnolence, which are expected events induced by the mechanism of zuranolone, serious AEs were not observed. However, it is noteworthy that the AEs induced by zuranolone were observed in the short term; further studies are needed to explore the incidence of AEs with long-term medication. In this context, studies regarding the concentrations of zuranolone in breast milk and its effects on infants are lacking. Therefore, further safety trials are needed to validate the use of zuranolone in PPD, especially during the lactation period.

Our study has certain limitations. Firstly, only six RCTs with 1884 patients were included and the sample size was insufficient (as demonstrated by TSA), further studies are therefore needed. Secondly, this study only compared the efficacy between zuranolone and placebo; the efficacy was not compared with that of other drugs acting on GABA_A_ receptors (such as brexanolone). In addition, all enrolled studies did not explore the efficacy of zuranolone co-initiated with other antidepressant agents. In this context, a phase III multicenter RCT comparing zuranolone plus an antidepressant drug to placebo is currently recruiting (NCT04476030). Thirdly, current studies mainly focus on the use of zuranolone in MDD and PPD; this limits generalization of the findings to mild and moderate depression. Finally, the long-term effectiveness of zuranolone in MDD remains unclear. In this context, Doesschate et al. suggested that zuranolone does not offer an advantage for the long-term treatment of MDD (TherapeuticsSageB, 2020). Further studies with adequate sample sizes will be needed to address these issues. Owing to the serious negative emotional impact of depression, the treatment of severe depression requires the use of medications that offer a rapid response. Zuranolone, which has a rapid onset of action, is popular in this regard. However, medications used for the treatment of depression also need to offer long-term and stable effects. Uncertainties may therefore exist until the long-term efficacy of zuranolone is validated.

## 5 Conclusion

The findings from this meta-analysis show that zuranolone has a rapid short-term positive effect on both PPD and MDD. They also suggest better efficacy with the 30 mg dose than other dosages. The absence of any reported deaths and withdraw rates among the participants, indicate that zuranolone is relatively safe. Although more TEAEs were observed in zuranolone, most of them were slight and temporary. However, further studies with larger sample sizes and longer durations of follow-up are needed to evaluate its efficacy and tolerability.

## Data Availability

The original contributions presented in the study are included in the article/Supplementary Material, further inquiries can be directed to the corresponding authors.
