# Supplementary material for: Efficacy and tolerability of zuranolone in patients with depression: a meta-analysis of randomized controlled trials
Source: Front Pharmacol. 2024 Jan 15;14:1334694. doi: 10.3389/fphar.2023.1334694 (PMC10823021; doi:10.3389/fphar.2023.1334694)
Supplement: Supplementary file 1 [file DataSheet1.ZIP › Supplementary Materials.doc]

**Efficacy and Tolerability of Zuranolone in Patients with Depression: A Meta-analysis of Randomized controlled trials**

Youjia Qiu^1, #^, Yuchen Tao^3, #^, Aojie Duan^1^, Xingzhou Wei^3^, Menghan Wang^3^, Minjia Xie^1^, Zhouqing Chen^1,*^, Jing Shang^2^, Zhong Wang^1, *^

*^1^* *Department of Neurosurgery & Brain and Nerve Research Laboratory,* *The First Affiliated Hospital of Soochow University, Suzhou, Jiangsu Province, 215006, China*

*^2^ Department of Psychiatry, The First Affiliated Hospital of Soochow University, Suzhou, Jiangsu Province, 215006, China*

*^3^ Suzhou Medical College of Soochow University, Suzhou, Jiangsu Province, 215002, China*

^#^ Youjia Qiu and Yuchen Tao contributed equally to this work.

^*^**Corresponding author**

Zhong Wang, Department of Neurosurgery & Brain and Nerve Research Laboratory, The First Affiliated Hospital of Soochow University, 188 Shizi Street, Suzhou, Jiangsu Province, 215006, China. Email address: [wangzhong761@163.com](mailto:wangzhong761@163.com).

Zhouqing Chen, Department of Neurosurgery, The First Affiliated Hospital of Soochow University, 188 Shizi Street, Suzhou, Jiangsu Province, 215006, China. Email address: zqchen6@163.com.

1. Table S1. Search result of included electronic database
2. Table S2. Inclusion, exclusion criteria, and outcome assessments of the included studies
3. Table S3. Risk of quality assessment (based on GRADE scale)
4. Table S4. Detailed information of AEs.
5. Figure S1. Forest plot for baseline HAMD score between zuranolone and placebo group
6. Figure S2: Forest plot for (A) headache; (B) dizziness; (C) nausea
7. Figure S3. Forest plot for (A) somnolence; (B)diarrhea; (C) sedation
8. Figure S4. Forest plot for (A) TEAE between 20 and 30 mg groups; (B) withdraw rates.
9. Figure S5. Forest plot for subgroup analysis in different dosages. (A) change of depressive severity score; (B) response; (C) remission
10. Figure S6. Forest plot for subgroup analysis in different types of depression.
11. Figure S7. Sensitive analysis of (A) change in depressive severity score; (B) response; (C) remission
12. Figure S8. Forest plot for subgroup analysis in different types of depression in response and remission
13. Figure S9. Trial sequential analysis of change of depressive severity score.
14. Figure S10. Penalized test of TSA based on change of depressive severity score.
15. Figure S11. PRISMA checklist

**Table S1: Detailed Search Strategy**

**PubMed**

| Search | Query | Results |
| --- | --- | --- |
| #1 | Depression [ MeSH Terms] | 626,581 |
| #2 | (Depressive Disorders[Title/Abstract]) OR (Disorder, Depressive[Title/Abstract]) OR (Disorders, Depressive[Title/Abstract]) OR (Neurosis, Depressive[Title/Abstract]) OR (Depressive Neuroses[Title/Abstract]) OR (Depressive Neurosis[Title/Abstract]) OR (Neuroses, Depressive[Title/Abstract]) OR (Depression, Endogenous[Title/Abstract]) OR (Depressions, Endogenous[Title/Abstract]) OR (Endogenous Depression[Title/Abstract]) OR (Endogenous Depressions[Title/Abstract]) OR (Depressive Syndrome[Title/Abstract]) OR (Depressive Syndromes[Title/Abstract]) OR (Syndrome, Depressive[Title/Abstract]) OR (Syndromes, Depressive[Title/Abstract]) OR (Depression, Neurotic[Title/Abstract]) OR (Depressions, Neurotic[Title/Abstract]) OR (Neurotic Depression[Title/Abstract]) OR (Neurotic Depressions[Title/Abstract]) OR (Melancholia[Title/Abstract]) OR (Melancholias[Title/Abstract]) OR (Unipolar Depression[Title/Abstract]) OR (Depression, Unipolar[Title/Abstract]) OR (Depressions, Unipolar[Title/Abstract]) OR (Unipolar Depressions[Title/Abstract]) | 7,433,620 |
| #3 | #1 OR #2 | 174,703 |
| #4 | "Zuranolone"[Mesh] | 59 |
| #5 | #3 AND #4 | 53 |

**Embase**

| Search | Query | Results |
| --- | --- | --- |
| #1 | 'Depression':ab,ti | 932,349 |
| #2 | 'Depressive Disorder':ab,ti | 111,818 |
| #3 | ' unipolar depression':ab,ti | 9163 |
| #4 | 'Symptom, Depressive':ab,ti | 28,185 |
| #5 | ' Emotional Depression ':ab,ti | 67,039 |
| #6 | #1 OR #2 OR #3 OR #4 OR #5 | 942,118 |
| #7 | ' zuranolone':ab,ti | 157 |
| #8 | 'SAGE-17':ab,ti | 3 |
| #9 | #7OR #8 | 157 |
| #10 | #6AND #9 | 142 |

**Cochrane**

| Search | Query | Results |
| --- | --- | --- |
| #1 | 'Depression':ab,ti | 768 |
| #2 | 'Depressive Disorder':ab,ti | 395 |
| #3 | ' unipolar depression':ab,ti | 9 |
| #4 | 'Symptom, Depressive':ab,ti | 431 |
| #5 | ' zuranolone':ab,ti | 31 |
| #6 | 'SAGE-17':ab,ti | 0 |
| #7 | (#1 OR #2 OR #3 OR #4) AND (#5OR #6) | 30 |

**Table S2: Meta-regression of different types of depression and dosages**

| **Effect modifier** | **ES** | **Coef** | **SE** | **P** | **95% LL** | **95% UL** |
| --- | --- | --- | --- | --- | --- | --- |
| **Change of depressive severity score** | | |  |  |  |  |
| Dosage | group | -0.0735 | 0.1025 | 0.501 | -0.3245 | 0.1775 |
|  | cons | -0.1817 | 0.2225 | 0.445 | -0.7262 | 0.3627 |
| Depression type | group | -0.2080 | 0.1662 | 0.257 | -0.6148 | 0.1987 |
|  | cons | -0.0617 | 0.2127 | 0.782 | -0.5822 | 0.4588 |
| **Response** |  |  |  |  |  |  |
| Dosage | group | 0.0713 | 0.1159 | 0.561 | -0.2123 | 0.3549 |
|  | cons | 0.1446 | 0.2575 | 0.595 | -0.4854 | 0.7747 |
| Depression type | group | 0.1719 | 0.1753 | 0.365 | -0.2570 | 0.6008 |
|  | cons | 0.0638 | 0.2334 | 0.794 | -0.5073 | 0.6349 |
| **Remission** |  |  |  |  |  |  |
| Dosage | group | 0.0003 | 0.1999 | 0.999 | -0.4891 | 0.4896 |
|  | cons | 0.4021 | 0.4438 | 0.400 | -0.6837 | 1.4879 |
| Depression type | group | 0.2766 | 0.2836 | 0.367 | -0.4174 | 0.9706 |
|  | cons | 0.3389 | 0.3722 | 0.930 | -0.8769 | 0.9447 |

**Table S3: Risk of quality assessment (based on GRADE scale)**

| Study | Year | Within-study bias | Reporting bias | Indirectness | Heterogeneity | Incoherence | Confidence rating | Reasons for downgrading |
| --- | --- | --- | --- | --- | --- | --- | --- | --- |
| Gundu-Brunce | 2019 | No concern | Low risk | No concern | Low risk | Low risk | High | [NA] |
| Clayton a | 2023 | No concern | Low risk | No concern | Low risk | Low risk | High | [NA] |
| Deligiannidis | 2021 | No concern | Low risk | No concern | Moderate | Low risk | High | [NA] |
| Kato | 2023 | No concern | Low risk | No concern | Low risk | Low risk | High | [NA] |
| Clayton b | 2023 | No concern | Low risk | No concern | Low risk | Low risk | High | [NA] |
| Deligiannidis | 2023 | No concern | Low risk | No concern | Low risk | Low risk | High | [NA] |

**Table S4. Detailed information of AEs**

| Study name | Year | AEs | Placebo | Zuranolone |
| --- | --- | --- | --- | --- |
| Clayton (20mg) | 2023 | Somnolence (19), headache (35), dizziness (21), sedation (17), nausea (19), diarrhea (21) | 54 | 78 |
| Clayton (30mg) | 2023 | Somnolence (21), headache (26), dizziness (18), sedation (15), nausea (16), diarrhea (22) | 54 | 64 |
| Clayton (50mg) | 2023 | Somnolence (49), headache (50), dizziness (43), sedation (21), nausea (9), diarrhea (9) | 48 | 133 |
| Deligiannidis | 2021 | Somnolence (20), headache (16), dizziness (10), nausea (9), diarrhea (7) | 29 | 37 |
| Deligiannidis | 2023 | Somnolence (31), headache (22), dizziness (43), sedation (4), nausea (11), diarrhea (11) | 40 | 62 |
| Gunduz-Bruce | 2019 | Somnolence (4), headache (15), dizziness (6), sedation (4), nausea (6), diarrhea (3) | 15 | 23 |
| Kato (20mg) | 2023 | Somnolence (15), dizziness (13); headache (9)  Sedation (1), gastrointestinal disorders (27) | 43 | 46 |
| Kato (30mg) | 2023 | Somnolence (22), dizziness (12); headache (8); gastrointestinal disorders (25) | 43 | 45 |

Figure S1. Forest plot for baseline HAMD-17 score between zuranolone and placebo group

Figure S2. Forest plot for (a) headache; (b) dizziness; (c) diarrhea

Figure S3. Forest plot for (a) somnolence; (b) sedation; (c) nausea

Figure S4. Forest plot for (a) TEAE between 20 and 30 mg groups; (b) withdraw rates.

Figure S5. Forest plot for subgroup analysis in different dosages. (a) change of depressive severity score; (b) response; (c) remission

Figure S6. Forest plot for subgroup analysis in different types of depression

Figure S7. Forest plot for subgroup analysis in different types of depression in response and remission

Figure S8. Sensitive analysis of (a) change in depressive severity score; (b) response; (c) remission

Figure S9. Trial sequential analysis of change of depressive severity score.

Figure S10. Penalized test of TSA based on change of depressive severity score.

Figure S11. PRISMA checklist
